# The OpenEar library of 3D models of the human temporal bone based on computed tomography and micro-slicing

**DOI:** 10.1038/sdata.2018.297

**Published:** 2019-01-08

**Authors:** Daniel Sieber, Peter Erfurt, Samuel John, Gabriel Ribeiro Dos Santos, Daniel Schurzig, Mads Sølvsten Sørensen, Thomas Lenarz

**Affiliations:** 1 MED-EL Elektromedizinische Geräte GesmbH, Innsbruck, Austria; 2 Hannover Medical School, Dept. Otorhinolaryngology, Hannover Germany; 3 Hörsys GmbH, Hannover, Germany; 4 Rigshospitalet, University of Copenhagen, Copenhagen, Denmark

**Keywords:** Surgery, Three-dimensional imaging, Computational models, Data acquisition, Sensory systems

## Abstract

Virtual reality surgical simulation of temporal bone surgery requires digitized models of the full anatomical region in high quality and colour information to allow realistic texturization. Existing datasets which are usually based on microCT imaging are unable to fulfil these requirements as per the limited specimen size, and lack of colour information. The OpenEar Dataset provides a library consisting of eight three-dimensional models of the human temporal bone to enable surgical training including colour data. Each dataset is based on a combination of multimodal imaging including Cone Beam Computed Tomography (CBCT) and micro-slicing. 3D reconstruction of micro-slicing images and subsequent registration to CBCT images allowed for relatively efficient multimodal segmentation of inner ear compartments, middle ear bones, tympanic membrane, relevant nerve structures, blood vessels and the temporal bone. Raw data from the experiment as well as voxel data and triangulated models from the segmentation are provided in full for use in surgical simulators or any other application which relies on high quality models of the human temporal bone.

## Background & Summary

Surgical interventions to treat hearing disorders, including hearing implants such as cochlear implants, middle ear implants or bone conduction implants require profound knowledge of the anatomy of the human temporal bone^1^. The level of experience of surgeons is therefore crucial to ensure optimal outcomes and low complication rates, necessitating extensive surgical training in preparation for ear surgery^2^. Until now, surgical training typically takes place in the operation theatres^3^ and in temporal bone laboratories, where surgeons are enabled to practice identification of anatomical landmarks on human cadaveric specimen^4,5^. However, in many places it has become increasingly difficult to obtain a sufficient number of temporal bones due to lack of availability because of ethical, cultural, regulatory and/or financial reasons.^6^ This situation led to the development of alternative training options such as artificial temporal bones from polymer materials^7–14^ or computer based surgical simulators, where haptic feedback devices are used in combination with advanced computer graphics to create a virtual reality training environment^15–19^. None of the mentioned systems is meant to entirely replace working with cadaveric specimen, but all of them offer a hazard free training environment which has the potential to be very effective in training surgeons at relatively low running costs. In fact, computer based surgical simulation allows the user to repeat procedures as often as desired while adding a new level of motivation for training by giving interactive feedback to the trainee. Recent studies^20–23^ have been able to demonstrate that with just two hours of self-directed simulator training a significant improvement of temporal bone dissection abilities measured in cadaveric specimen can be demonstrated in entry level surgeons.

Most of the currently available simulation systems are limited by the fact that they are based on low-quality datasets. Those datasets are acquired by Computed Tomography (CT) which results in low detail models and lack of information on soft tissue structures such as membranes. micro CT imaging-based data might be able to overcome the lack of details, however current Micro CT machines only allow scanning of small specimen and it is therefore impossible to model a sufficiently big portion of the anatomy for surgical simulation. For both CT and micro CT based datasets there is no colour information available and so texturing of models for simulation is performed manually resulting in quite unnatural colouring. The Visible Ear Simulator (VES) freeware^19^ available at https://ves.alexandra.dk is currently the only simulator providing higher fidelity simulation as it is based on naturally coloured cryosection data^24^ and 3D rendered at 125 voxels/mm^3^. On the other hand, as the creation and processing of cryosection data is extremely laborious, over more than ten years the VES has been limited to only one anatomy which limits the learning experience by neglecting the significant learning potential of inter individual variability of anatomy^25–27^.

**Figure 1:**
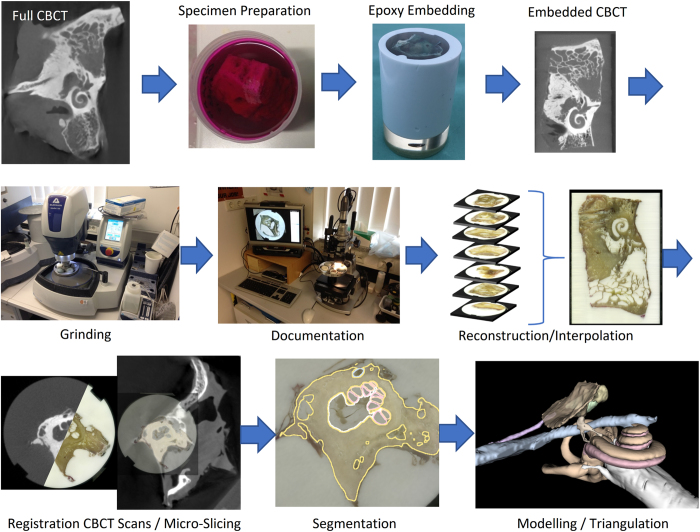
Overview of processes used in creation of the OpenEar Dataset.

The aim of the OpenEar project was therefore to develop a new approach and method which allows for much more efficient creation of high fidelity coloured models of the human temporal bone. Such an efficient method was found in the combination of imaging using ionizing radiation and micro-slicing of cadaveric human temporal bone specimens. Both imaging modalities were three dimensionally reconstructed, registered and segmented. An overview of all process steps is provided in Fig. 1. As a result, raster/voxel imaging data of eight digitized human temporal bones is provided, including segmentations of all relevant anatomical structures and triangulated 3D models thereof. The entire dataset is provided to the public in full to allow for surgical training or research relying on high-quality models of the human ear such as navigated/robotic surgery, development of automated segmentation algorithms and finite-element simulations of the middle and inner ear mechanics.

The OpenEar represents a valuable addition to the datasets which had previously been available to the field of surgical simulation. Compared to datasets created using clinical CT imaging, the OpenEar dataset provides a much higher level of detail, particularly when it comes to delicate soft tissue structures such as the tympanic membrane or basilar membrane. In contrast to datasets based on micro CT imaging, the OpenEar dataset is able to provide a sufficient model size to cover all aspects of middle and inner ear surgery and comparable to temporal bone specimen used in clinical education courses.

Like the Visible Ear dataset, the OpenEar dataset provides high fidelity volumetric geometry data including colour information which enables surgical simulators to be textured based on the actual colours of the anatomical specimen. This allows to overcome the need to rely on manual false colour texturing used in simulations based on data from CT/micro CT imaging. The Visible Ear dataset continues to provide an excellent quality temporal bone model for surgical simulation, while the OpenEar dataset enriches the learning experience by introducing the significant factor of anatomical variation in eight unique human ears.

## Methods

Eight fresh human temporal bone specimens from four adult subjects were used to create this dataset. Temporal bones were kindly donated by the Institute of Pathology of the Hannover Medical School. They were obtained from patients who contributed their corpses to medical education and research. As the specimens were used anonymously, no approval by the institutional ethical board was necessary.

### Specimen Preparation

Specimens were scanned using a 3D ACCUITOMO 170 Digital Cone Beam Computed Tomography (CBCT) scanner (J. MORITA TOKYO MFG. CORP., Japan) within a period of one hour after specimen sampling. Resulting CT images were reconstructed and exported as DICOM using the i-Dixel software (J. MORITA TOKYO MFG. CORP., Japan) with a voxel size of 0.250 mm.

After CBCT imaging, specimens were cut to fit an embedding mould and fully immersed in a fixation solution of 4% Formol in phosphate buffered saline (PBS). To allow for better penetration of the fixation solution and further process fluids into the inner ear lumina, a small opening was drilled into the superior semi-circular canal, and fixation solution was applied to the superior semi-circular canal using a syringe and cannula to optimally fix the intracochlear structures. After 72 h of storage in the fixation solution, specimens were rinsed in PBS to remove Formol from the specimens. The specimens were dehydrated in four steps by sequential immersion in 70, 90 and 100% Ethanol and afterwards 100% Methanol for 2 days each. For improved contrast of the soft tissue structures against the embedding epoxy, 0.1% Acid Fuchsin was added during the Ethanol steps. Afterwards, specimens were dried at room temperature in a fume hood for about one hour. Finally, the specimens were embedded in epoxy resin (SPECI-FIX 40, STRUERS, Denmark) to immobilize and preserve any mobile structures inside the temporal bone like membranes or the ossicular chain during further process steps. To improve penetration of the epoxy resin into the smaller lumina of the temporal bone, the embedded specimens were put in a vacuum desiccator. Embedded specimens were cured at room temperature for at least 7 days before continuing with further process steps.

After embedding, all specimens were scanned again using CBCT, to also capture the outer geometry of the epoxy overmould to be used for referencing the different imaging modalities. Scanning mode was set to ‘HiRes’ this time, scanning parameters were set to 80 kV tube voltage, 2 mA tube current, 360° scanning angle and 60x60mm Field of View (FOV). Reconstruction and DICOM export were performed in the same manner as before mentioned, but at a 0.125 mm voxel size.

### Micro-Slicing

Micro-slicing was performed by sequential grinding and microscopic documentation of the specimens. Grinding was performed using an AutoMet250 Grinder-Polisher (BUEHLER, Lake Bluff, IL, USA) equipped with silicon carbide (SIC) grinding paper with a Grit of P 800. After each removal, the novel layer was documented using a VHX-2000 measurement microscope (KEYENCE Corporation, Osaka, Japan) equipped with a VH-Z20UR zoom lens at 20x magnification mode using image stitching functionality. The effective removal was determined by measuring the height of the remaining overmould using a micrometre gauge.

Alignment of the images from micro-slicing as a prerequisite step for reconstruction of the image stack, was performed by custom made software using Python scripting and the packages of the Anaconda collection (Continuum Analytics, Austin, TX, US). A template matching technique was implemented to align the images along the overmould outline which served as reference geometry due to its relative invariance across images. A brute force search approach was first used to find the approximate translation and rotation of the overmould outline in the image, followed by a local optimization method to find the best translation, rotation, shear and scale of the overmould in the image.

Due to the relatively large size of the images (~3700×3700 pixels) the computational effort to perform such operation makes it unattractive to perform the computation on the central processing units (CPUs) of modern personal computers. Instead it was decided to implement the described operation on a graphics processing unit (GPU) which allows for a massive parallelization of the required computations.

### Reconstruction/Registration

The slice thickness in histological sections is inhomogeneous as per the unavoidable technical tolerances of the used grinding process. To be able to use the acquired images with existing software for processing of medical three-dimensional images, slices had to be homogenized. The aligned images were therefore interpolated to a homogeneous slice thickness of 150 μm using a virtual image stack. The original images were put on the positions in the virtual image stack, which came closest to their original position to keep interpolation related losses in image quality minimal. Those positions in the stack which did not have an original image assigned to them, were then linearly interpolated between the nearest neighbouring original images.

Reconstruction of the micro-slicing image stack was performed using 3D Slicer 4.8.0 (http://www.slicer.org). The data from CBCT imaging was also imported into 3D Slicer as DICOM series. After a rough manual alignment of the high-resolution embedded CBCT scan with the micro-slicing images, 3D Slicer’s built-in BRAINSfit algorithm was used to automatically register both imaging modalities. In a second step, the low-resolution, larger CBCT scan of the unembedded temporal bone was registered to the high-resolution CBCT scan using a manual landmark-based registration, followed by the automatic BRAINSfit registration algorithm. After completion of the two registrations mentioned, it became possible to display all three imaging modalities, the large portion low-resolution CBCT, the high resolution embedded CBCT and the reconstructed micro-slicing data aligned in parallel.

### Segmentation

A threshold supported manual paint segmentation technique was chosen for segmentation of the datasets using 3D Slicer. Small structures were segmented using the high resolution embedded CBCT dataset to have access to the best possible image quality. Larger structures were segmented using the larger low resolution unembedded CBCT scan to be able to model the biggest possible portion of the temporal bone. Multimodal overlay visualization was utilized in those anatomic structures which are partially delimited by bony structures and partially by soft tissue structures. Table 1 gives an overview of the anatomical structures and imaging modalities used for segmentation of these structures.

### 3D Surface Model Creation

Segmented volumes were converted to three-dimensional triangulated surface models of all anatomical structures. The bone segment was additionally exported as a voxel model as per the requirements of surgical simulators. Triangulation was performed in 3D Slicer and included moderate smoothing during model creation. Finally, the mesh quality and complexity were optimized using free software tools Graphite, Meshlab and Meshmixer (Autodesk, San Rafael, CA, USA).

### Step-by-Step Process Descriptions

Further details on the processes used to create the OpenEar dataset including step-by-step description of all process steps are included in Supplementary File 1 of this Data Descriptor.

### Code Availability

All custom-made software which was created to align and interpolate micro-slicing data is available in full and without restrictions at the Zenodo open access research data repository^28,29^.

All software tools used for the postprocessing of image data are either freeware or Open Source where code can be accessed via the individual software project websites.

3D Slicer is available at https://www.slicer.org/

Meshlab is available at http://www.meshlab.net/

Graphite is available at http://alice.loria.fr/software/graphite/

Meshmixer is available at http://www.meshmixer.com/

## Data Records

As a result of the described process we are able to provide three-dimensionally reconstructed, co-registered sets of CBCT scans and micro-slicing colour images of the human temporal bone. Eight digitized human temporal bones were uploaded to the Zenodo open access research data repository and are available for download free of charge through a permanent Digital Object Identifier (DOI) (Data Citation 1). They may be shared, used and adapted even for commercial use with the requirement of attributing to this original work as per a Creative Commons Attribution 4.0 License.

Volume datasets from all imaging modalities performed in this project are provided as 3D Nearly Raw Raster Data (NRRD) images in the final data processing stage. CBCT images are provided as isotropic voxel data at a resolution of 125 μm. To allow access to the higher in-plane resolution of those images, micro-slicing data is provided at an inhomogeneous 50×50×150 μm voxel resolution. Coordinate directions are invariant across all imaging modalities provided. Additionally, all raw data from the experiment, including CT scans in DICOM format and micro-slicing images stored as TIFF files are provided to enable the community to work with the original data and eventually improve the above described reconstruction, registration and segmentation processes.

Segmentations of scala tympani, scala vestibuli, malleus, incus, stapes, facial nerve, chorda tympani, tympanic membrane, external auditory canal, sigmoid sinus and dura, carotid artery and bone are provided at a 125 μm isotropic voxel resolution as 3D NRRD images and can be viewed with any of the co-registered imaging modalities in the background. Additionally, triangulated models of those segments are provided with an optimized mesh quality using about 70 vertices/mm^2^ surface area in Polygon File Format (PLY) format which can also easily be converted to the common STL file format using 3D Slicer (e.g. for 3D printing).

The data repository contains a compressed subfolder for each of the datasets labelled with the specimen identifier. The eight temporal bone datasets were labelled Alpha, Beta, Gamma, Delta, Epsilon, Zeta, Eta and Theta. The compressed subfolder for each specimen contains the data as described in Table 2.

## Technical Validation

The numerical representation of the human temporal bone provided in this work is limited by its nature to only be able reproduce the colour and structures found in temporal bone specimens ex-vivo and post-mortem. These may differ from the colours and structures found intraoperatively in patients in-vivo, despite all efforts to process specimen immediately after becoming available.

Furthermore, several of the process steps in the creation of data may result in certain technical inaccuracies:

- Dehydration of specimens during preparation for embedding may result in shrinkage of certain soft tissue structures in the embedded datasets. E.g. the facial nerve may shrink and no longer fill out its bony canal entirely
- Mechanical forces applied for fixation of the embedded specimens in the grinding machine holder may result in geometric deformations of the specimens in the micro-slicing data
- The stitching of images as performed with the optical microscope may lead to minor inaccuracies in the optical image acquisition in the micro-slicing data
- The measurement of remaining overmould height after each grinding step in micro-slicing involves a certain measurement error particularly in a somewhat elastic specimen
- 3D Reconstruction of the micro-slicing data relies on algorithmic identification of the outline of the specimens embedding as well as the overmould height measurement determining the position of each slice in the image stack. Above mentioned inaccuracies in the experimental setup as well as numerical errors may result in reconstruction inaccuracies.
- Interpolation of the reconstructed micro slicing data to create a homogeneously spaced image stack may result in certain image artefacts.
- Registration of the micro-slicing and computed tomography images is based on the identical accurate imaging representation in both modalities. As per the above described inaccuracies of both the 3D micro-slicing and CT image modalities, as well as due to numerical errors in the registration process, inaccuracies may occur.

Regarding the computed tomography imaging, Lund *et.al*.^30^ found that the accuracy of the ACCUITOMO CBCT device used in this experiment can be assumed to be below the voxel size of 0.125 mm.

**Figure 2:**
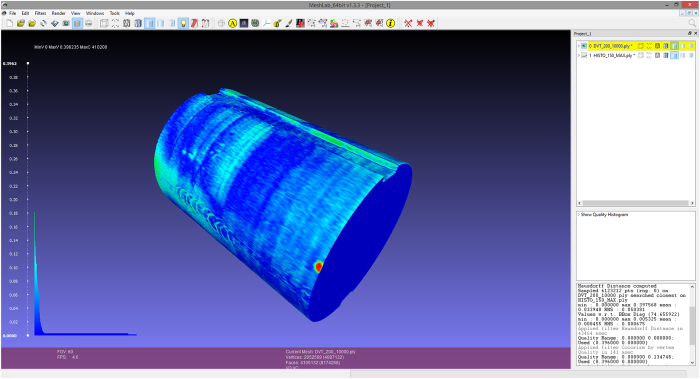
Visualization of differences between CBCT and micro-slicing data for the Zeta dataset using the Meshlab Hausdorff distance analysis.

Quantification of the overall accuracy of the micro-slicing data proves more difficult due to the high number of interconnected process steps and influencing factors, and the possible inhomogeneity of inaccuracies within the volume. A comparison of volumetric and geometric properties of the final product from acquisition, reconstruction and registration of micro-slicing with the CBCT as reference was chosen as most viable option. Threshold based segmentations of the epoxy overmould outline in the CBCT and micro-slicing modalities were analytically compared using the Meshlab Hausdorff distance filter^31^ yielding the mean and maximum geometric error between CBCT and micro-slicing. Figure 2 exemplary shows the visualization of the Hausdorff distance analysis and histogram of error for one dataset. The Meshlab Compute Geometric Measures function allowed for the calculation of the volumetric error of the micro-slicing image related to the CBCT images. Colour coded is the distance between the surface of reconstructed overmold geometry from micro-slicing and CBCT imaging. Visible error/deformations of the epoxy overmold include e.g. screw indentations from mounting the specimen in the grinding machine holder in different positions.

Results for the geometric and volumetric errors for each of the datasets are summarized in Table 3. The described analysis could unfortunately not be performed for dataset Eta, due to an artefact in the CBCT acquisition which made segmenting the overmould outline impossible.

High fidelity surgical simulators currently operate at a voxel resolution of about 125 voxels/mm^3^^19^ and a corresponding isotropic pixel size of about 200 μm. The above described accuracies of the CBCT and the micro-slicing datasets are therefore considered to be acceptable as they lie within the pixel resolution of the computer simulation which they were primarily created for. However, the OpenEar dataset may be limited for applications requiring higher levels of detail and accuracy such as e.g. cochlear microanatomy.

## Usage Notes

The OpenEar datasets allow for an entirely new view on the anatomy of the human temporal bone. The co-registration of reconstructed CBCT and micro-slicing data allows for viewing two-dimensional slices of the ear seamless and/or simultaneous in radiation based and optical modalities. When looking at a mid-modiolar section of the inner ear, for instance, the CBCT image will give the viewer a solid understanding of where the bony constraints of the inner ear space are located and allow for semi-automatic threshold-based segmentation techniques. Being able to amend or change to the information from the micro-slicing images, on the other hand, allows for a more complete picture and more precise segmentations as also soft tissue structures like e.g. the basilar membrane or tympanic membrane can easily be located.

For viewing of the data, 3D Slicer is the recommended free software package which was also used during the creation of the datasets, however there are numerous software options to work with TIFF, DICOM or NRRD data which may be considered.

**Figure 3:**
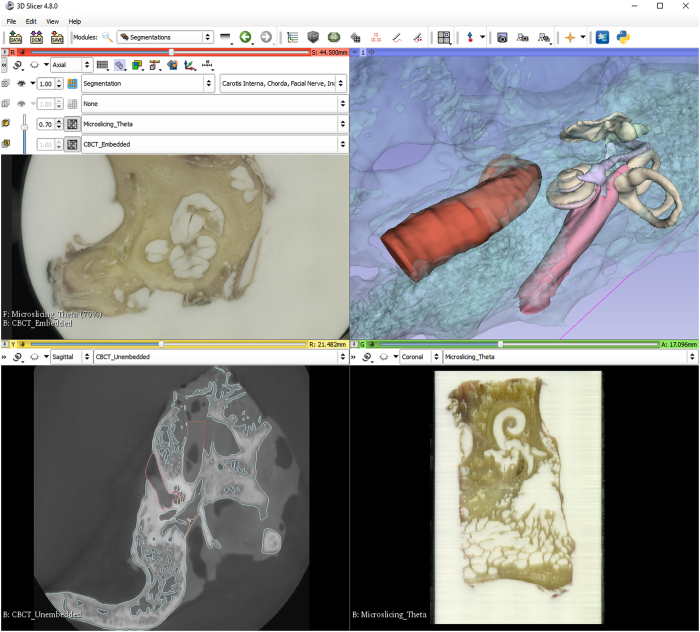
3D Slicer visualization of different imaging modalities of the Theta dataset. (**a**) Image fusion slice with 30% CBCT and 70% micro-slicing. (**b**) Three-dimensional reconstruction of anatomical segments from delineation. (**c**) CBCT slice with anatomy segmentation delineation overlay. (**d**) Reconstructed slice from micro-slicing.

When using 3D Slicer to work with the datasets, it is recommended to load the reconstructed micro-slicing dataset, as well as the registered 3D Slicer volumes of the CBCT unembedded and embedded specimen and the segmentation file. Figure 3 shows how to use the ‘Slice Viewer’ controls, to view two image volumes simultaneously as foreground and background layer, as well as the segmentation overlay and 3D representation of the geometric segmentation.

## Additional information

**How to cite this article**: Sieber, D. *et al*. The OpenEar library of 3D models of the human temporal bone based on computed tomography and micro-slicing. *Sci. Data*. 6:180297 doi: 10.1038/sdata.2018.297 (2019).

**Publisher’s note**: Springer Nature remains neutral with regard to jurisdictional claims in published maps and institutional affiliations.

## Supplementary Material



Supplementary Information

## Figures and Tables

**Table 1:** Image modality used for segmentation of different anatomical structures.

Segment	Micro-Slicing	CBCT Embedded Specimen	CBCT Full Specimen
Scala Tympani/Vestibuli	X	X	
Malleus/Incus/Stapes		X	
Facial Nerve/Chorda		X	
Tympanic Membrane/EAC	X		X
Sinus/Carotis Interna			X
Bone			X

**Table 2:** Data content per compressed specimen subfolder (e.g. Alpha).

Steps	Description of files	File format
CBCT Unembedded	Folders containing DICOM series of files representing a CBCT scan of the unembedded temporal bone specimen right after specimen sampling (not available for ALPHA and BETA)	DICOM (.dcm)
CBCT Embedded	Folders containing DICOM series of files representing a CBCT scan of the specimen after cutting, dying, drying and embedding into the epoxy overmould	DICOM (.dcm)
Microslicing Raw	Series of images from micro-slicing grinding process, each image documents the embedded specimen after removal of material using the grinding machine	TIFF (.tiff)
Reconstruction Microslicing	3D reconstructed colour datasets from micro-slicing after alignment of slices and interpolation to a homogenous spaced image stack as volumetric voxel dataTransformations: Comma separated value table where each row contains the nine entries of a 3x3 transformation matrix of one micro-slicing image from the stack in C-like/row-major order	NRRD (.nrrd)CSV (.csv)
	Materials for Reconstruction:Target images containing the outline of the epoxy overmould used for alignment of the images from micro-slicingLayer Positions: Comma separated value table with the information on the position of each micro-slicing image in the stack as described by its distance to the initial slices	GIMP/PNG/SVG(.xcf)/(.png)/(.svg)CSV (.csv)
Registred Slicer Volumes	3D reconstructed grayscale datasets from CBCT @ 125 μm resolution as volumetric voxel data• CBCT_Unembedded: Unembedded CBCT registered to align with micro-slicing• CBCT_Embedded: Embedded CBCT registered to align with micro-slicing, resized to the coordinate space size of the unembedded CBCT	NRRD (.nrrd)NRRD (.nrrd)
	Transformations:Transformation matrices used to register datasets to each otherAccuracy assessment: (not available for ETA)Meshlab screenshot of the Hausdorff analysisText file with results from geometric and volumetric analysis3D Slicer segmentation file delineating overmould in CBCT and micro-slicing	.H5 (.h5)PNG (.png)TXT (.txt)NRRD (.nrrd)
Segmentation	3D Slicer segmentation file assigning voxels of the registred CBCT volumes to one of the anatomical structures delineated	NRRD (.nrrd)
3D Models	Triangulated, smoothed 3D models of all anatomical structures as segmented in 3D Slicer after mesh quality optimization@ ~70 vertices / mm^2^Bone volume as volumetric voxel data	PLY (.ply)NRRD (.nrrd)

**Table 3:** Geometric and volumetric error for datasets of the OpenEar library.

Dataset	Geometric error [mm]		Volumetric error [%]
	Mean	Max	
Alpha	0.064	0.736	0.349
Beta	0.090	0.951	0.929
Gamma	0.082	0.734	0.753
Delta	0.087	0.627	0.646
Epsilon	0.056	0.529	0.296
Zeta	0.034	0.398	0.370
Eta	—	—	—
Theta	0.043	0.288	0.128
