# Supplementary material for: The OpenEar library of 3D models of the human temporal bone based on computed tomography and micro-slicing
Source: Sci Data. 2019 Jan 8;6:180297. doi: 10.1038/sdata.2018.297 (PMC6326113; doi:10.1038/sdata.2018.297)
Supplement: Supplementary Information [file sdata2018297-s2.pdf]

Supplementary Information:
Step-by-Step Protocols of OpenEar Creation Process

Table of Contents

Protocol of micro slicing of epoxy embedded temporal bone specimen……….. 2

Protocol of creation of target image from image stack………………………………. 4

Protocol of alignment and interpolation of micro slicing data…………………..… 6

Protocol of reconstruction / registration of micro slicing data…………………….. 9

Protocol of segmentation and modelling of anatomy…………………………….… 13

Protocol of micro slicing of epoxy embedded temporal bone specimen

Aim

The aim of this process step is to acquire two dimensional images of an epoxy embedded temporal bone specimen at as many positions along the long axis of the specimen as possible. This is achieved by repeated grinding off a defined layer thickness from the specimen, and subsequent imaging by 2D optical microscopy. The layer thickness chosen is a compromise between the level of detail to be obtained and the time it takes to process specimen. Processing several specimens at the same time may improve the efficiency of the process but add risk of errors/mixup during the process.

Materials

- Buehler AutoMet250 with custom made specimen holder

- CarbiMet P1200 self-adhesive sand paper

- Keyence VHX-2000 with VH-Z20UR lens

- Micrometer DIN863 Set for 10-70mm

- Pressure air

Time planning

After a certain learning curve, about seven minutes per slice were needed

Protocol

Setup Phase

1. Setup Microscope
   - Initialize X/Y Stage and Autofocus “Automatically”
   - Adjust “Camera Settings”
     - Shutter: 1/60 sec
     - Gain: 0 dB
     - White Balance: Use the “Push Set” option placing and focusing a piece of white paper at approx. the same operating distance as the specimen
2. Setup Grinding Machine
   - Adjust Settings
     - Depth: 0.2 mm
     - Pressure: “ZAXIS”, 45N
     - Probe RPM: 40
     - Disc RPM: 500
     - Mode: “COMP”
     - Check in “WATER” Menu that “STAGE” is set to “ON”
     - Mount specimen in specimen holder with minimal hand torque
3. Setup Micrometer Screw

- Use probes to ensure calibration is ok
- Mount micrometer to allow for comfortable working

Slicing Phase

Before starting slicing, please make sure that everything that you need to perform the steps below is ready for many hours of work. Keep in mind that the following steps will be of destructive nature and cannot be reversed. A CBCT scan has to therefore be performed before starting the slicing process. To avoid deformations of the specimen mounting them in the specimen holder the epoxy embedding should have hardened at least 1-2 weeks.

1. Mount the specimen into the specimen holder and tighten screws carefully to avoid deformation of the specimen. In case of not using all slots in the specimen holder, use dummy epoxy overmolds to avoid any unbalanced mass.
2. Attach specimen holder to grinding machine
3. Start grinding machine
4. After completion of grinding dry the specimens
    - Dry specimen holder and specimens roughly using cotton blanket
    - Dry specimens surface and edges carefully using paper towel
    - Clean surface of specimens using pressure air
    - Visually check surfaces for remaining particles and remove if any are found
5. Make sure microscope's X/Y table is centered
6. Position the specimen holder horizontally under microscope
7. Align specimen 1 to be roughly concentric with the light source of the microscope
8. Perform autofocus on specimen 1
9. Check result of autofocus procedure visually
10. Set borders of 2D image stitching functionality
11. Start 2D image stitching
12. Replace sandpaper on grinding machine while waiting for image stitching
13. Save image file for specimen 1 (Naming: 000.TIF, 001.TIF, 002.TIF and so on…)
14. Repeat steps 5-13 (not step 12) for specimen 2 and all further specimen accordingly
15. Measure remaining specimens height for all specimen and document in excel sheet
16. Check after every slice that enough specimen is sticking out of the specimen holder. Reposition the specimen when needed and carefully tighten screws at lowest possible torque to avoid deformation of specimen
17. Repeat steps 2-16 until the entire temporal bone is gone, or it becomes impossible to further advance and reposition the specimens in the specimen holder

Protocol of creation of target from image stack

Aim

The aim of this process step is to create a target image which can later be used to align all images from a micro slicing dataset. The alignment is performed by identifying and aligning the outline of the epoxy overmold in all images of the dataset. Therefore, a target image which is reduced to only contain the epoxy overmold outline information has to be created.

Materials

- WACOM Pen Digitizer Tablet Small (Model CTL-480 was used here)
- Gnu Image Manipulation Program (GIMP) version 2.8
- "Offset Path" script by user "RobA" available from http://www.silent9.com/incoming/scripts/ (version 2013-04-24)
- Micro slicing images, see "Protocol of micro slicing of epoxy embedded temporal bone specimen"

Installation

- Install GIMP
- Copy the offset_path.scm script into the GIMP scripts folder where all other '.scm' files are located

Protocol

1. Choose an image from the micro slicing
   - Preferably from a central position in the image stack
   - Try to choose a slice with minimal defects in the overmold outline
     - No air bubbles
     - No dust/grinding particles
     - No major deformations
2. Load the respective .tif file from the micro slicing to GIMP2
3. In the "Import from TIFF" dialog choose to import "Page 1"
4. Go to "Layer" -> "Transparency" -> "Add Alpha Channel" to add a transparency variable per pixel
5. Go to "Layer" -> "Duplicate Layer" to add a backup up of the original image
6. Go to "Tools" -> "Path" to activate the Paths module
7. Increase Zoom factor to 400%
8. Create a path along the outline of the overmold outline by left clicking on it using the Wacom digitizer; To correct mistaken points use "Strg+Shift" to delete them and draw a new point
9. To close the path after drawing the last point hold the "Strg" key and click on the first point again
10. Rename the path just created to "Outline"
11. Right click on the Outline path in the Paths Dialog and choose Offset Paths to run the script by RobA. Create and rename two new paths, one which is bigger and one which is smaller than the original outline. An offset of 20-50 pixels symmetric in both directions has proven to be good. For histologies with more deformation 50 is the better choice, for histologies with no visible outline deformation 20 seems the best choice. To get a "one fits all" process we

ended up using a two-step process with a 50 target to search the rough outline, and a 20 target to find the precise outline.

12. Check the offset outline paths for points which were not correctly transformed. Such points can be moved by drag and drop or deleted using Strg+Shift.
13. Select the smaller outline and go to “Select” -> “From path”,
14. Go to “Select” -> “Feather” and choose a feathering of 5px
15. Go to “Edit” -> “Cut”. You should now see everything inside of the inner outline checkerboard style
16. Select the larger outline and go to “Select” -> “From path”
17. Go to “Select” -> “Invert”, feather as before, cut as before
18. Go to “Image” -> “Autocrop Image”
19. Now you may add rotation to the target. Rotating the target may help to overcome false minima in the search operation to find the outline in the image. These come from the fact that the original unrotated target is not interpolated, whereas all the other transformations of the target are interpolated pictures. So the unrotated image might be considered the best fit, just because it is not interpolated and results in a false low penalty function result. Rotating the target by 45° makes sure that all transformed picture undergo the same interpolation degradation eliminating the error source. In a two-step process the second step target must be vertical oriented again. To add rotation do the following:
    a. Go to “Tools” -> “Transform Tools” -> “Rotate” and rotate the image 45° (Make sure the Layer is selected in the Layers Dialog and in the “Tool Options” there is also “Layers” selected
    b. Right-click on the Outline path and select “Duplicate Path”
    c. Rename the new path to “Outline rotated”
    d. Switch “Tool Options” to “Path”, and use the rotate tool again to rotate the “Outline rotated” path by 45°
20. Go to “Image” -> “Canvas Size” and enter an odd number of pixels for both directions (e.g. 3701x3701px), click on “Center” in the “Offset” dialog
21. Export the target image by going to “File” -> “Export As”, set File Type to “PNG Image (*.png)”
22. In the Export dialog deactivate “Save background color” and “save color values from transparent pixels”
23. Export the path by right clicking on Outline rotated and selecting “Export Path”, use .SVG as filesuffix
24. Save your entire work by clicking “File” -> “Save as”

Protocol of alignment and interpolation of micro slicing data

Aim

The aim of this process step is to align all images of a micro slicing dataset by alignment along the epoxy overmold outline and subsequent interpolation of the images to allow 3D reconstruction of the dataset. Image alignment is performed by combination of a constrained brute search global optimization algorithm with a subsequent local optimization to find the transformation between target image and search image. Interpolation is performed using a virtual image stack which is filled with the acquired images from micro slicing and in which any slices which remain empty are then interpolated from the surrounding images.

Materials

- Micro slicing images, see "Protocol of micro slicing of epoxy embedded temporal bone specimen"
- Protocol of specimen height progression, see "Protocol of micro slicing of epoxy embedded temporal bone specimen"
- Target Image, see "Protocol of creation of target from image stack"
- Anaconda Python distribution plus several packages as listed below
- Python scripts and iPython Notebooks from Zenodo/Github

Installation

- Make sure to install an up-to-date GPU driver and have an OpenCL compatible GPU
- Install an up to date browser e.g. Firefox
- Install Anaconda (Version used was Anaconda 3 2.5.0 64bit)
- Install Anaconda packages (many downloadable from Christoph Gohlke's web page) using the "pip install" function:
    - Appdirs (version used was 1.4.0)
    - Pytools (version used was 2016.1)
    - Pyopencl (version used was 2015.2.4)
    - Svg.path (version used was 2.1.1)
- Download the versions of "pattern_finder_gpu" and "reconstruct_volume_from_RGB_slices" used to create the published dataset from Zenodo:
  https://doi.org/10.5281/zenodo.1344923
  https://doi.org/10.5281/zenodo.1400785
  Check the Github displayed on the respective Zenodo pages to check for potential updated versions on Github.

Protocol

Image alignment

1. Launch Anacondas "jupyter" GUI and open the "reconstruct_volume_from_RGB_slices.ipynb" notebook from the repository to perform image stack alignment

2. Scroll down to the “Start of main script” section and adjust the paths to the situation on your PC:
    - Set the path to the target image .PNG file
      (See “Protocol of creation of target from image stack”)
    - Set the path to the target outline .SVG file
      (See “Protocol of creation of target from image stack”)
    - Set the path to the folder containing all .TIF files from one micro slicing series
      (See “Protocol of micro slicing of epoxy embedded temporal bone specimen”)
3. In the “Definition of Search Strategy” adjust settings of the alignment
    - Rescale: Value for downsampling of the image (bigger than 0 and smaller or equal to 1= full res). To save computation time reduce this value, to increase accuracy increase this value.
    - Angle Range to search in during brute force optimization. Format: (Start_Angle, End_Angle, No of steps in between), Choose the search range large enough to be confident to be able to find the most rotated image of the stack. Subsequent steps will build on the best position found in previous steps, so search range is symmetrical to zero typically. Set number of steps sufficiently high to get a smooth curve plot for rotation vs. error value for plot = True or plot=’all’ as shown below

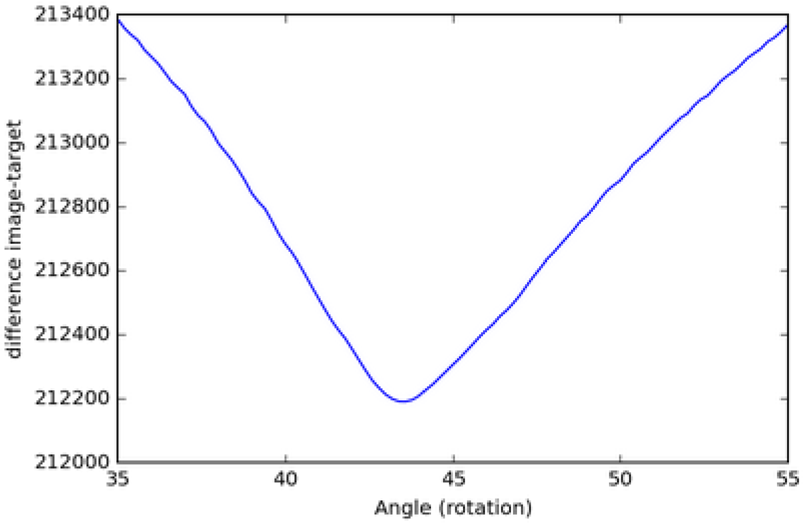

    - ROI Size for brute force optimization. Make sure the ROI is large enough, to allow for finding of the pattern in image. Check the heat plot in plot=’all’ to make sure it looks somewhat like below. Note that rescaling between steps can make a larger ROI necessary even though the image is already aligned pretty well from previous steps.

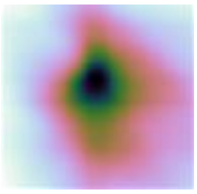

    - Tolerance in local optimization: The higher the value, the faster the computation, the lower the value, the more accurate the result
4. In the “Execution of Image Alignment” section, adjust parameters to the situation on your PC:

- Set the write_files variable to the path that aligned images shall be written to. Set it to 'False' if you do not want to write files to disc
- Set the plot variable to "False", "True" or "all" to set the level of feedback you want to get from the optimization process

5. In jupyter choose "Cell"->"Run All" from the dropdown menu
6. The computation will now be performed for every image of the image stack. This can take several minutes to several days, depending on the settings chosen above. If errors occur, try to debug. If unsuccessful, contact the authors of the code (Daniel Sieber, Samuel John).

<u>Image Interpolation</u>

7. Next is the interpolation of the micro slicing images to create a homogeneously distanced image stack as required by most medical 3D viewers. As a prerequisite you need to export the positions of the layers from micro slicing as a comma separated value (CSV) list. If you used Excel to document slices, create a new tab and copy/recalculate your slice position data such, that every row represents one layer, with the first layer located at 0 representing the position of 000.TIF, and all further rows representing further layers and their distance to the first layer. Select all the rows in the column you created and use "Save as" to save as CSV and make sure that only the tab you just created is saved when asked.
8. Launch Anacondas "jupyter" GUI and open the "image_stack_interpolation.ipynb" notebook from the repository
9. Scroll down to "Definition of constants" and adjust the values/paths:
    - targetPixelNumXY: Sets the size to which every image is resampled to in X and Y direction, represented by two values separated by a comma; the higher the values chosen, the bigger the data volume created , the lower the values chosen the worse the image resolution
    - targetLayerSize: Sets the distance between slices in Z direction. Creates a homogenously spaced virtual image stack where the layers are spaced according to the value set.
    - csvfile: path to the .CSV file containing the positional data on the micro slicing slices as described in 7.)
    - images: path to the micro slicing images plus asterix based file name extension to create an image collection for interpolation of the images
    - savedir: path which shall be used to store the images of the virtual image stack containing the micro slicing images amended by interpolated images in the empty positions in between

10. In jupyter choose "Cell"->"Run All" from the dropdown menu
11. The computation will now be performed and the original and interpolated images written to disc. This can take several minutes to hours, depending on the settings chosen above. If errors occur, try to debug. If unsuccessful, contact the author of the code (Daniel Sieber).

Protocol of reconstruction / registration of micro slicing data

Aim

The aim of this process step is to import the aligned and interpolated image stack from micro slicing into 3D Slicer and to reconstruct the dataset in three dimensions. In a second step the reconstructed dataset is registered to the corresponding CBCT datasets to allow seamless simultaneous use of the aligned datasets in further process steps.

Materials

- Aligned and interpolated micro slicing images, see “Protocol of alignment and interpolation of micro slicing data”
- CT scans of the unembedded and epoxy embedded specimen before micro slicing
- 3D Slicer multi-platform free medical imaging and computation software (version used was 4.8.0)
- Meshmixer software (version 11.0.544 was used here)
- Meshlab software (version 1.3.3 was used here)

Installation

- Install 3D Slicer software
- Install Meshmixer
- Install Meshlab

Protocol

<u>Alignment</u>

1. Use the “Load Data” Module, go to the folder where the interpolated micro slicing slices are located and select the first image of the image stack. Activate the “Show Options” checkbox and make sure that the “Single File” check box is deactivated
2. Use the “Volumes” Module, go to “Active Volume” and rename the volume to a meaningful name like “HISTO”, go to “Volume Information” and set the image spacing to the values which were used in interpolation, see “Protocol of alignment and interpolation of micro slicing data”
3. Check the “IJK to RAS Direction Matrix”. In case the first and second entry on the diagonal are negative, means that the X any Y coordinates are flipped. As this may result in issues later, it should be fixed now:
    1. Use the “Transforms” Module, go to “Active Transform” and create a new linear Transform
    2. Use “Rename current node” to name the transform “FlipXY”
    3. Under “Edit” invert the first and second diagonal entry of the “Transform Matrix”
    4. Under “Apply Transform”, apply the FlipXY transform to the HISTO dataset and push the “Harden Transform” button to make the change permanent.
4. In case you want to use the micro slicing in more than one resolution or an anisotropic/isotropic version, steps 1-3 have to be repeated for all datasets
5. Use the “DICOM” module to import and load the CBCT scan of the embedded specimen, and rename it to a useful name like CBCT

6. In the slice view visualization options set CBCT as foreground and HISTO as background dataset and adjust the visibility such that both datasets will be shown 50%/50% (although not yet aligned)
7. Use the “Transforms” module to create a new linear transform, give it a useful name e.g. CBCT_InitialGuess, apply the transformation to the CBCT dataset(s) using the “Apply Transformation” section, adjust the “translation” sliders in the “edit” section until the two datasets fit roughly onto each other. In case coordinates seem flipped, invert the signs of the main diagonal entries of the transform matrix. Harden the transformation in the “Apply Transformation” section when done.
8. Use the “Vector to Scalar Volume” module (under Converters) to create a scalar copy of the HISTO dataset, rename the new dataset to e.g. HISTO_Scalar
9. Use the “General Registration (BRAINS)” module (under Registration) and execute a new Registration with the following settings:
    - Fixed Volume: HISTO_Scalar
    - Moving Volume: CBCT
    - Percentage of Samples: 0.2 (run it at 0.002 first to see if it runs properly)
    - Slicer Linear Transform: Create a new one called CBCT_BrainsFit
    - Initialization Transform: None
    - Choose “Rigid 6DOF”
10. Use the “Transforms” module to harden the CBCT_Brainsfit transformation for the CBCT dataset
11. Use the “Volumes” module to rename the CBCT volume to CBCT_Registred
12. Use the “DICOM” module to import and load the CBCT scan of the unembedded specimen
13. Use the “Volumes” module to rename to a useful name like CBCTBig
14. Use the “Landmark Registration” module (under Registration) to roughly align the big and small CBCT scans with the following settings:
    1. Fixed Image: CBCT_Registred
    2. Moving Image: CBCTBig
    3. Use the “Add Landmark” feature to add three landmarks and identify them in both scans, for example:
        i. Apex
        ii. Center of RW
        iii. End of Malleus handle
    4. Registration Type: Affine Registration
    5. Registration Mode: Rigid (in some cases Rigid+Scale yields better results)
15. Use the “Transforms” module to rename the “Transform” transformation from the landmark registration to CBCTBig_InitialGuess, apply it to the CBCTBig volume and harden the transform
16. Use the “General Registration (BRAINS)” module and create a new Registration with the following settings:
    1. Fixed Volume: CBCT_Registred
    2. Moving Volume: CBCTBig
    3. Percentage of Samples: 0.2 (run it at 0.002 first to see if it runs properly)
    4. Output Slicer Linear Transform: Create a new one called CBCTBig_BrainsFit
    5. Initialization Transform: None

6. Choose “Rigid 6DOF”
17. Use the “Transforms” module and harden the CBCTBig_Brainsfit transformation for the CBCTBig dataset(s)
18. Use the “Crop Volume” module (from Converters) to crop and resample the largest image of the scene:
    1. Input Volume: CBCTBig_Registred
    2. Input ROI: Create new Annotation Region of Interest (ROI)
    3. Change ROI size such that all relevant structures are included
    4. Use Interpolated cropping, interpolation option:
        i. Isotropic output voxel
        ii. Scaling factor: 0.5 (To upsample from 250µm to 125µm resolution)
        iii. Linear interpolation
19. Use the “Volumes” module to rename the CBCTBig-subvolume-scale0.5 volume to CBCTBig_Registred
20. Use the “Resample Image (BRAINS)” module (from Registration) to resample the CBCT_registred volume to have the exact same size and resolution as the CBCTBig_Registred volume for later segmentation:
    1. Image to Warp: CBCT_Registred
    2. Reference: CBCTBig_Registred
    3. Output Image: Create new image CBCT_Registred_FullKOS
    4. Pixel Type: Short
    5. Default value: Minimum Value of the CBCT_registred from Volumes module
21. Use the “Save File” widget to save your work

Accuracy Check

Two accuracy check measurements have been implemented for the datasets. The goal is to measure the geometric and volumetric errors in the micro slicing data in relation to the CBCT data.

Geometric error

The Hausdorff distances from one dataset to another is the distance between one voxel of the dataset and its closest voxel on the other dataset. In our case, the measured set is the outline of the epoxy mold of the micro-slicing data with the target being the same outline from the CBCT data.

1. Create a new Segmentation using the “Segment Editor” Module
2. First choose the CBCT_Registred_FullKOS dataset under “Master Volume”
3. Use the “Threshold” Effect and adjust the maximum value to the highest possible value, while setting the lower threshold to capture the entire epoxy overmold without getting too much noise like artifacts outside the epoxy mold
4. Now choose the HISTO_Scalar volume under “Master Volume” and use threshold effect as in 3.)
5. Use the “Export Models” function in the “Segmentations” module to export the segmentation, creating a new model hierarchy

6. Use the “Save File” widget to save the models over the overmold, select the .ply format
7. The models are then imported in *Meshmixer* to remove all the inner surfaces such as only the outer surface of the mold remains. The mesh operations used to do this in Meshmixer are Select (S), Replace (R), Remove (X), Invert Selection (I), and Fill (F).
8. Import the results from Meshmixer in Meshlab
9. The accuracy measurement is done by measuring the Hausdorff distance between the two models (under Filters/Sampling/Hausdorff distance).
10. The models are then colorized by Vertex Quality (Color Creation and processing/ Colorize by Vertex Quality) and a Quality Histogram is created (Render/Show Quality Histogram).

<u>Volumetric Error</u>

The Volumetric Error measurement is also based on the epoxy overmold segmentations from the micro-slicing and CBCT data. It gives the volume ratio between the subset of voxels contained in only one of the two sets and the CBCT set.

1. The 3DSlicer outline segments from the previous measurement are used again, the inner holes are filled using the *Scissors* tool. In the “Segment Editor” module a new segment named *Merged Error* is created. Using the *Logical operators* tool, the error between the two segments is computed using the following operations: (voxels of HISTO – voxels of CBXT) + (voxels of CBCT – voxels of HISTO). The newly created segment represents exclusive *or* between the CBCT and HISTO segments. This means it contains all the voxels that are only in one of the two sets.
2. The CBCT and Merged Error segments are now exported as ply models in analogy to steps 5./6. of the geometric error computation.
3. Import the two segments in *Meshlab*. For both model, the mesh volume is then calculated using *Filters/Quality Measure and Computations/ Compute Geometric Measures.* The volumetric error calculates as the volume of Merged Error divided by the volume of CBCT mesh.

Protocol of segmentation and modelling of anatomy

Aim

The aim of this process step is to delineate the different anatomical structures in the reconstructed and registered micro slicing and CBCT datasets, to allow subsequent creation of three-dimensional models of these anatomical structures. This is done by manual and in some cases, threshold supported segmentation techniques followed by an automatic triangulation of the segmented geometries.

Materials

- WACOM Pen Digitizer Tablet Small (Model CTL-480 was used here)
- 3D Slicer latest version (version 4.8.0 was used here)
- Reconstructed and Registered datasets, see "Protocol of reconstruction and registration of micro slicing data"
- Graphite software (version 3-1 Release 2015 was used here)
- Meshmixer software (version 11.0.544 was used here)
- Meshlab software (version 1.3.3 was used here)

Installation

- Install latest version of 3D Slicer
- Install the latest version of Graphite
- Install the latest version of Meshmixer
- Install the latest version of Mashlab

Protocol

<u>Segmentation</u>

Use the "Load Data" module in 3DSlicer to load the HISTO, CBCT_Registered_FullKOS and CBCTBig_Registred datasets, see "Protocol of reconstruction and registration of micro slicing data". Segmentation is performed using the "Segment Editor" module. In the slice view visualization options set one of the CBCT datasets as foreground and HISTO as background dataset and adjust the visibility to show one of the datasets, or both datasets simultaneously. Which setting to use is dependent on the anatomical segment to be deliniated. Most of the segmentation can be done using the CBCT data. Micro-slicing data is mainly used to identify fine bony structures (e.g. stapes) and soft tissue structures (e.g. tympanic membrane, basilar membrane) and to help finding landmarks (e.g. junction between Facial Nerve and Chorda Tympani).

As an initial step the bone threshold value (BTV) is determined, all voxels with an intensity higher than BTV will be considered as bone. The BTV is chosen such that most of the air cells partition are kept while no artifact due to soft tissue is added on the surface of the bone. The value must be manually adjusted while segmenting, until the resulting segmentations have an adequate size.

The segments were created in the following order, using the "Add new empty segment" function:

1) Scala Tympani, Scala Vestibuli, Ossicles, Facial Nerve, Chorda and Carotis

The “Paint” tool is the main tool used to do the segmentation in these segments and the Digitizer Pen is used to control the brush. It is important to always check that two segments are not overlapping by setting the editable area to “Outside all segments”. Activating the “Editable Intensity Range” allows the user to set a range of voxel values outside of which the tool does not work. For the bony structures, this range is from the BTV to the max value. For cavities and other structures, the range is from the min value to the BTV. The “Scissors” tool is used to create clean cuts on the outer ends of ducts of nerve structures and the Carotid artery. To check the results of segmentation and optimize them, the “Show 3D” function can be used, this is particularly helpful for surface optimization and small structures like the Stapes.

2) Tympanic Membrane

The membrane segment is created using the micro-slicing data. First, the segment is painted on the slices where the image is not interpolated (so it’s an original picture of the sample). The gaps between each painted slice are then filled using the “Fill between Slices” tool. The remaining holes are then filled directly on the 3D window using the “Paint” effect with the “Sphere brush” function activated.

3) Bone

The bone segment is obtained using the “Threshold” effect (From the BTV to the max value). The “Keep largest island” function from the “Islands” effect is used to discard undesired and unconnected regions of the segmentation. The “Scissors” effect is used to remove any possible artifact. The integrity of the bone surfaces is checked and holes on surfaces which should be closed are manually closed using a sphere brush eventually combined with the “Editable Intensity Range” function and gradually reducing the lower value below the BTV value until a smooth surface is achieved.

In normal temporal bone specimen, the ossicles are not be embedded in bone and the Facial Nerve should be surrounded by a bony canal. This can be checked using the “Show 3D” function and if necessary the bone segment must be modified using “Paint” and “Erase”.

4) External Auditory Canal

Before segmenting the EAC, the holes between the Tympanic membrane and the Bone must be filled so that the junction is completely seamless. The EAC segment is then created using the Paint tool. The Scissors effect is used to obtain a clean cut at the outer end of the EAC.

5) Sinus-Dura

A large sphere brush (e.g. Size 20) is used to roughly obtain a first version of the segment representing Sinus and Dura. The “Keep the Largest Island” operation is used to remove unconnected islands. At this point, a lot of the air cells are still filled with Dura. The goal of the next steps is to remove it. The “Margin/Shrink” operation is applied with a 3mm margin for example. This operation can take several minutes and aims to separate the parts of the segment that are in the air cells from the main island. “Keep the Largest island” is then used again. Most of the air cells if not all are empty at this point. For the main island to fit the bone again, it is necessary to use the Margin/Grow operation with the same margin value as for the Shrink operation. It lasts also several minutes. The outer border of this segment must be cleaned using Scissors such as clean cuts are obtained and that the Sinus and Dura are hidden behind the bone from the surgeons pre-operative viewpoint.

Modelling

Once it has made been sure that all segments are acceptable in the 3D view and not overlapping, the models are exported using the “Segmentations” module (Operation : Export, Output type : Models) and subsequently saved as .ply files using the “Save Data” Widget. The Bone segment is exported as a Labelmap (Operation : Export, Output Type : Labelmap) and subsequently saved as .nrrd file using the “Save Data” Widget.

1) Repairing and Remeshing the models

The steps described in this paragraph are applied for all exported models except for the bone. Performing these steps will greatly improve the mesh quality and reduce complexity of the models.

1) Start the Graphite software
2) Use File->Load to load the ply file from Slicer
3) Use the Surface->Repair->repair surface operation with the following parameters
    a. Epsilon: 0,1
    b. Min comp area: 0,03
    c. Max hole area: 1e-3
    d. Max hole edges: 2000
    e. Max degree3
    f. dist: 0,0
4) Use the the Surface->Repair->merge vertices operation with the following parameter
    a. Epsilon: 0,01
5) Remesh the model, this means creating a new mesh based on the former one with a new amount of points and improved mesh quality. The operation is Surface->Remesh->remesh smooth with the following parameters:

    a. Nb points : See table below
    b. Tri shape adapt : 1.0
    c. Tri size adapt : 0.0)

Segment	Stapes	Incus, Malleus	Chorda Tympani	Tympanic Membrane	Scala Tympani, CochleoVestibular Nerve	Carotis, Facial Nerve, Scala vestibuli, External Auditory Canal	Sinus-Dura
Nb points	1000	2000	3000	4000	8000	10000	50000

This should result in a relatively homogenous mesh element size. If this is not the case, the number of points may have to be adjusted.

Once the operation is complete, the newly created remeshed model is exported using the File->save current function.

If needed, open the remeshed model in Meshmixer to fix remaining defects of the surface by replacing them using a smoothed surface.

In the MeshLab software, import the remeshed models. Sometimes the imported models appear with a completely dark surface. In this case the surface normal are falsely inverted, this can be corrected by applying the Filters->Normals, Curvatures and Orientation-> invert face orientation. The model is then saved again.
